# Violence in rural areas against disabled people from the perspective of their families

**DOI:** 10.1590/0034-7167-2022-0404

**Published:** 2023-05-29

**Authors:** Carmem Layana Jadischke Bandeira, Jaqueline Arboit, Fernanda Honnef, Ethel Bastos da Silva, Andressa de Andrade, Marta Cocco da Costa

**Affiliations:** I Universidade Federal de Santa Maria. Palmeira das Missões, Rio Grande do Sul, Brazil

**Keywords:** Violence, Rural Health, Disabled Persons, Nursing, Family, Violencia, Salud Rural, Personas con Discapacidad, Enfermería, Familia, Violência, Saúde da População Rural, Pessoas com Deficiência, Enfermagem, Família

## Abstract

**Objectives:**

to know the violence spoken and felt by disabled people, living in rural areas, from the perspective of their families.

**Methods:**

a descriptive-exploratory and qualitative study, carried out in four municipalities in Rio Grande do Sul, Brazil. Twelve family members who lived with disabled people in rural areas participated. Data were collected through semi-structured interviews and analyzed using thematic content analysis.

**Results:**

disabled people, living in rural areas, experienced physical, psychological and sexual violence, perpetrated by family members, colleagues, community members and health professionals. Adaptations were mentioned in family dynamics for the care of disabled people, social, financial and leisure impacts, and challenges in access and accessibility to education and health services.

**Final Considerations:**

violence against this population manifests itself in a reality with socioeconomic and family particularities, marked by exclusion, disrespect and denial of rights and access to fundamental goods and services.

## RESUMO

**Objetivos:**

conhecer a violência falada e sentida pelas pessoas com deficiência, residentes em áreas rurais, na perspectiva de seus familiares.

**Métodos:**

estudo descritivo-exploratório de abordagem qualitativa, desenvolvido em quatro municípios do Rio Grande do Sul, Brasil. Participaram 12 familiares que residiam com pessoas com deficiência em áreas rurais. Os dados foram coletados por entrevistas semiestruturadas, sendo analisados pela análise de conteúdo temática.

**Resultados:**

as pessoas com deficiência, residentes em áreas rurais, vivenciaram violência física, psicológica e sexual, perpetrada por familiares, colegas, pessoas da comunidade e profissionais de saúde. Foram mencionadas adaptações nas dinâmicas familiares para o cuidado da pessoa com deficiência, impactos sociais, financeiros e no lazer, e desafios no acesso e acessibilidade a serviços de educação e saúde.

**Considerações Finais:**

a violência contra essa população manifesta-se em uma realidade com particularidades socioeconômicas e familiares, marcada pela exclusão, desrespeito e negação de direitos e acesso a bens e serviços fundamentais.

## RESUMEN

**Objetivos:**

conocer la violencia hablada y sentida por personas con discapacidad, que viven en zonas rurales, desde la perspectiva de sus familias.

**Métodos:**

estudio descriptivo-exploratorio con abordaje cualitativo, realizado en cuatro municipios de Rio Grande do Sul, Brasil. Participaron 12 familiares que vivían con personas con discapacidad en zonas rurales. Los datos fueron recolectados a través de entrevistas semiestructuradas y analizados mediante análisis de contenido temático.

**Resultados:**

las personas con discapacidad, residentes en áreas rurales, experimentaron violencia física, psicológica y sexual, perpetrada por familiares, colegas, miembros de la comunidad y profesionales de la salud. Se mencionaron adaptaciones en la dinámica familiar para el cuidado de personas con discapacidad, impactos sociales, económicos y de ocio, y desafíos en el acceso y accesibilidad a los servicios de educación y salud.

**Consideraciones Finales:**

la violencia contra esta población se manifiesta en una realidad con particularidades socioeconómicas y familiares, marcada por la exclusión, el irrespeto y la negación de derechos y acceso a bienes y servicios fundamentales.

## INTRODUCTION

Violence is a social and health problem, as it threatens the development of communities, affecting the quality of life of millions of people^(1)^. It is a complex and multicausal phenomenon to which everyone is susceptible, considering disabled people (DP) who live in rural areas^(2)^. In the case of violence against DP, it is characterized by any action or omission, practiced in a public or private place, that causes death, damage or physical or psychological suffering^(3)^.

**Figure 1:**
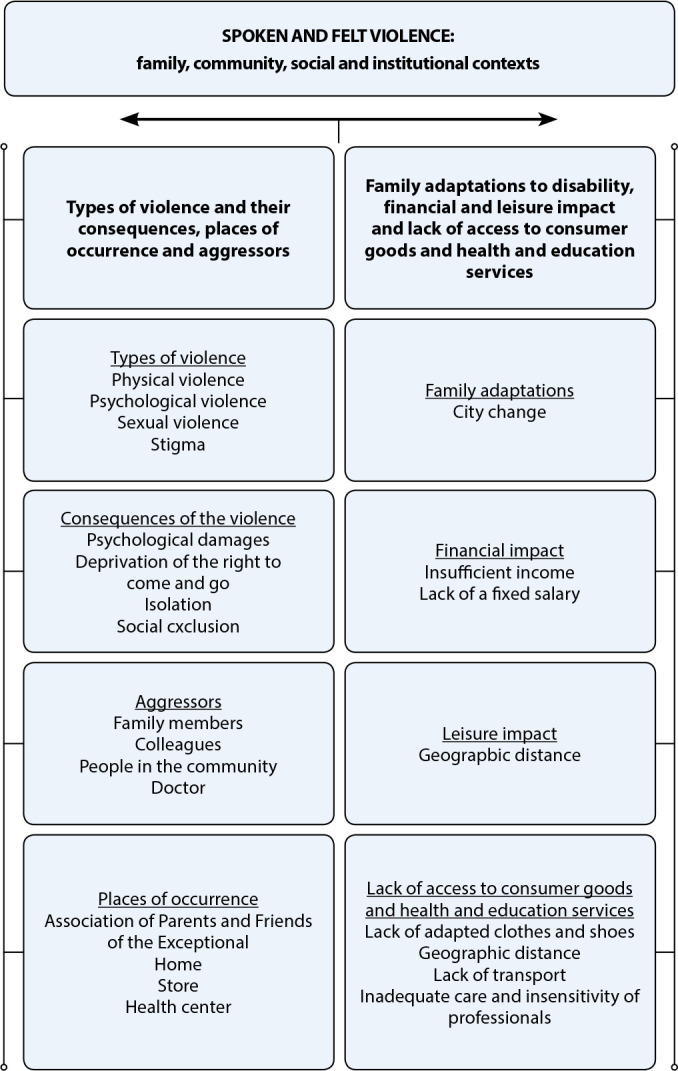
Coding tree

For this study, the concept of DP was adopted, according to which these are people who have long-term physical, mental, intellectual, or sensory impairments that, when coexisting with one or more obstacles, may hinder their full and effective participation in society on an equal basis with other individuals. This concept considers a biopsychosocial assessment of disability that encompasses the interaction between various elements, such as changes in body functions and structures, socio-environmental, psychological and personal components, and restriction in carrying out activities and participation^(3)^.

According to the Brazilian National Health Survey, carried out in 2019, 17.3 million people aged two years or older had some type of visual, hearing, mental or physical impairment, of which 14.4 million lived in urban households and 2.9 million, in rural households^(4)^. The prevalence of each type of disability was: 3.4% (visual); 1.1% (auditory); 6.5% (physical); and 1.2% (intellectual)^(4)^.

Regarding violence perpetrated against DP, the most reported type of violence was physical violence (53%), followed by psychological violence (32%) and neglect/abandonment (30%)^(5)^. In rural areas, a study showed that 68% of DP experienced some type of psychological violence, and 58%, physical violence^(2)^. Such violence has considerable repercussions on the lives of DP, as demonstrated by a study that examined the relationship of abuse with psychological and physical health outcomes in adults with developmental disabilities, which pointed out that childhood disability-related abuse and adult mixed abuse were significantly related to psychological (depression, post-traumatic stress disorder) and physical health problems^(6)^.

The rural context encompasses a diversity of people, with their cultures and way of life, from which individuals see themselves and the world around them. This context is not isolated, but intertwined with specifics of life and work^(7)^. People residing in rural areas tend to face numerous barriers, compared to those residing in urban areas, even considering the population with similar socioeconomic characteristics. Among these barriers, there are precarious access to health services^(8-9)^, lack of transport and geographic distances^(8)^.

Thus, national and international literature highlight growing disparities between urban and rural populations, which are exacerbated for DP residing in rural areas^(10)^. The combination of disability with poverty and contextual deprivation, for example, makes them vulnerable to various forms of discrimination, impacting their ability to access basic services^(11)^. In this sense, social, environmental and physical barriers are reported that prevent the full implementation of policies that defend the rights of DP in rural areas^(12)^, since this population suffers from unemployment^(10,13-15)^, restricted access to health services, which are limited in these areas^(13)^, having inaccessible facilities and inadequate equipment^(14)^.

In addition to the denial of rights, the grievances generated by violence make it difficult to experience fully human and social equality. The persistence and multiplicity of forms of expression of violence, throughout history, indicate the importance of the theme and the need to investigate how this practice interferes in the life process of those who suffer it^(16-19)^.

Given the above, this study sought to hear DP family members regarding the violence spoken and felt by the latter. It is notable that the field of study on violence against DP, specifically in rural areas, is incipient and sometimes only considers factors, such as prevalence and factors associated with violence, disregarding other elements that have an interface with the problem. Based on this interpretation, this investigation considers the context and cultural, social, family, political and institutional aspects that affect the experience of violence experienced by DP, from the point of view of their families. With this, the family is recognized as a dynamic unit, made up of individuals united by blood ties, of interest or affection that, together, build a history. Still, they have unique values, knowledge, practices and beliefs, establishing interactions with each other and with other people at different levels, in a given social, cultural and political context, influencing this space and being influenced by it^(20)^.

In this context, there is a need to give visibility to the theme, as society seeks to suppress the stigma that DP are less capable, enabling their active participation, guaranteeing rights and equal conditions with other people. Considering the exposed problem, we sought to answer the following guiding question: what are the situations of spoken and felt violence experienced by DP residing in rural areas, from the perspective of their families?

## OBJECTIVES

To know the violence spoken and felt by DP, living in rural areas, from the perspective of their families.

## METHODS

### Ethical aspects

The recommendations of Resolution 466/2012 of the Brazilian National Health Council were followed, which provides for research with human beings^(21)^. Thus, data collection began after approval by the Research Ethics Committee. Participants were informed about the objectives, risks and benefits of the investigation, by reading and explaining the Informed Consent Form (ICF), which was signed by participants in two copies. To ensure their anonymity, the letter P, for participant, and the respective number were used in the sequence of the interviews (e.g., P1, P2, P3…P12).

### Theoretical framework

For this study, we used the theoretical frameworks of violence^(1,3)^, rurality^(7)^ and family^(20)^.

### Study design

This is a qualitative research^(22)^, descriptive-exploratory^(23)^, guided by the COnsolidated criteria for REporting Qualitative research (COREQ)^(24)^.

### Methodological procedures

#### Study setting

The investigation was carried out in four municipalities in the North and Northwest of Rio Grande do Sul, Brazil, belonging to the 2^nd^ and 15^th^ Regional Health Coordinations. These Coordinations together account for 52 municipalities, predominantly rural and with a Gross Domestic Product per capita of R$25,000 (US$5,000.00) to R$29,999 (US$6,000.00) and a Socioeconomic Development Index between 0.740 and 0.779 and ≥ 0.780, respectively^(25)^. The aforementioned regions are part of a matrix project scenario entitled “*Determinantes Sociais de Saúde em Pessoas com Deficiência, Famílias e Rede de Apoio no Cenário Rural: múltiplas vulnerabilidades*”.

The choice of the four cities in the study scenario is justified by having more than 70% of the population living in rural areas. In addition, these had the highest prevalence of violence against DP, according to previous data from the quantitative part of the matrix project, which sought to analyze the prevalence of situations of violence perpetrated against DP in these municipalities.

#### Data source

Participants were captured from the results of the matrix project, constituting a convenience sample. This information supported the construction of this research, seeking the perspective of family members of DP who experienced some type of violence. Participants were a representative of each DP family residing in rural areas, totaling 12 family members. The inclusion criteria used were being of legal age, residing with DP and having the cognitive conditions to respond to the interview. The exclusion criterion was being absent from the residence on the day of collection.

#### Data collection and organization

Prior to data production, a meeting was organized with the nurse responsible for the Family Health Strategy of each municipality, to facilitate the data collection stage. Based on this, through the Community Health Workers (CHW) in rural areas, prior telephone appointments were made for interviews with each family member. On the scheduled date, the researchers, together with the CHW from the area of ascription of each family, went to their homes. Thus, the first contact of the researchers with the possible participants took place in person.

Data were collected from December 2018 to February 2019, using a semi-structured interview^(22)^, through a script built by the researchers responsible, containing closed-ended questions for the sociodemographic and disability characterization and open-ended questions related to the object of study. Two pilot tests were carried out, with subsequent script adaptation, aiming at a better understanding of the questions by participants. The data from the tests were discarded.

The interviews were carried out by two researchers with experience in the data collection technique and theme. Prior to this, they introduced themselves to potential participants, mentioning their professional training, link with the higher education institution and trajectory in the research, reading and explaining the ICF. All family members invited to participate in the study accepted, and there were no dropouts. The interviews took place at home, only in the presence of participant and researchers, in order to guarantee privacy and protect the ethical principles of research. They were audio-recorded (MP3), with an average duration of 40 minutes. As for the number of participants, the end of fieldwork was determined when the internal logic of the object of study was understood^(26)^.

Among the questions for sociodemographic characterization, participants were asked about their age, race, education and family ties with DP. They were also asked about the type of disability. As for the open questions, some questions asked to guide the interview were: what is violence against DP in rural areas? What types of violence against DP are there? Have DP residing in this family ever suffered violence?

#### Data analysis

The material from the interviews was submitted to thematic content analysis, in three stages: pre-analysis, material exploration and treatment of results obtained and interpretation^(22)^. First, the data obtained from interview recordings were literally transcribed in a text editor program, constituting the research corpus. Afterwards, in the pre-analysis stage, the recordings were listened to and the transcribed material was skipped reading, emerging the initial impressions. Subsequently, through repeated readings, excerpts from statements were highlighted in different colors based on the ideas contained in them. Using chromatic technique allowed composing the material for further analysis. In the material exploration stage, the common information found in the content was selected, allowing to list the meaning cores, which are words, phrases and expressions that give meaning to this content. Afterwards, the nuclei of meaning were added to define the category and subcategories.

The last stage consisted in treatment of results and interpretation, in which the researchers made inferences and interpretations about the results, considering the objective of this study. Thus, the results were discussed with national and international scientific literature related to the topic.

## RESULTS

As for participant characterization, most were female, eight women and four men, with a mean age of 49 years. Regarding kinship with DP, four were parents; four were spouses; two were nephews(nieces); one was a son; and one was sibling. As for race, nine self-declared white and three, brown. With regard to education, eight had incomplete elementary education; two had completed elementary school; one had incomplete high school; and one had completed high school. Regarding the type of disability presented by DP, seven had an intellectual disability, and five had a physical disability.

From thematic analysis, a category emerged, *SPOKEN AND FELT VIOLENCE: family, community, social and institutional contexts*. This was broken down into two subcategories: *Types of violence and their consequences, places of occurrence and aggressors*; and *Family adaptations to disability, financial and leisure impact and lack of access to consumer goods and health and education services*.

### SPOKEN AND FELT VIOLENCE: family, community, social and institutional contexts

This category has two subcategories. The first presents the types of violence present in the lives of DP residing in rural areas, places where they occurred, aggressors and consequences for DP and their families. The second describes the dynamics of DP families regarding adaptations to disability, impact on the financial sphere, on social and leisure relationships and on access to goods and services.

### Types of violence and their consequences, places of occurrence and aggressors

Among the types of violence mentioned by family members of DP, there are situations of physical violence that have had negative consequences in the lives of those involved, including psychological damages and deprivation of the right to come and go. This violence manifests itself in different ways and in different spaces of society.

*Arriving* [from Association of Parents and Friends of the Exceptional (APAE - *Associação de Pais e Amigos dos Excepcionais)*] *the daughter scratched, leg running blood, scratched, as if it were made of blade or wire* [...] *she never wanted to go again, she was afraid to go.* (P2)

*She left the house and wanted to go with her daughter* [...] *then, there, they beat her, she ran away and came away* [...] *sometimes she goes up to a day without showering. Then her daughter beat her up because of it.* (P7)

Physical violence is also present in conflicting family relationships. This distances DP from their family members, subjecting them to being under the responsibility of people with less proximity.

*She has two more brothers, but she doesn’t like it there. They said that, when we are very tired, then we should take them to stay there for a while with the brothers, but she doesn’t want to, because her daughter hit her.* (P7)

Psychological violence in DPs’ lives manifested itself in the form of nicknames given, even by family members. In this direction, participants explained the “pains” caused by such nicknames.

*What I hear most is nickname. Also, within the family there are people who say something* [...] *we don’t even like to talk* [...]. (P6)

*I see people “putting down” the person* [referring to the use of nicknames] [...] *the one there “has a disability”, is “crippled”* [...] *it hurts our hearts* [...]. (P11)

The stigma against DP was evidenced by the disparate views of people in living with this population group, in community and social spaces. Some participants reported experiencing embarrassment and rejection by DP, having as one of the consequences the isolation and social exclusion of this and the whole family, which deprived themselves of leisure activities that would provide social and community coexistence.

*People made fun of him* [...] *they saw him fall and people made fun of him. At first it was quite embarrassing for us, and then, when he started using crutches, a lot of people made fun of him.* (P1)

[People] *are afraid of them* [referring to DP] [...] *in a store in a city nearby, I asked to give him the bathroom and they didn’t want to; he peed his pants, I had to buy clothes to wear him. And once we went to a wedding and because of disability they didn’t want to sit at the table with us. I don’t like to go to parties because there are ignorant people and they look at them.* (P3)

[...] *when we go out, we take her to the doctor, people keep looking at her* [...] *at the health center, they laugh when I take her to appointments, because she has a funny legs.* (P9)

*If she goes out like that and her leg isn’t covered, they look at you with a weird look.* (P12)

DP also experience situations of sexual violence, with a case perpetrated by a health professional during an appointment being mentioned.

[...] *she has a problem with her head and not her body, and the doctor, in a consultation, started to feel her breasts, ordered her blouse and bra to be taken off, and she felt irritated* [...] *I said, “Doctor, what does her body have to do with her problem?” She got very angry, she left there in that agitation* [...]. (P9)

From this subcategory, it is observed that DP in rural areas experienced situations of physical, psychological and sexual violence that, according to family members, contributed negatively to their health and well-being.

### Family adaptations to disability, financial and leisure impact and lack of access to consumer goods and health and education services

Participants reported having undergone adaptations of family life dynamics in order to meet DP’s care needs.

*We didn’t even know how to deal with him because he had so many injuries. He lived in the city, so they moved to the countryside, we had to adapt to live with him, live with his problem and work.* (P11)

Participants reported financial impacts for the family, considering that the amount of the Continuous Cash Benefit Program (BPC - *Benefício de Prestação Continuada*) received by DP was not enough. This was aggravated because, in rural areas, most families base their economy on family farming, with no fixed wages. Despite this, growing food and raising animals for their own consumption helped to alleviate this difficulty.

*The income is not enough* [...] *it is lacking, sometimes, right* [...] *it’s just that we, in the colony, plant to eat manioc, beans, then it’s enough.* (P3)

[...] *last month we had to do everything on our own, then it didn’t arrive* [referring to the money] *we have other earnings that we can pay, but if it was just hers, it wouldn’t come.* (P4)

*In fact, if you’re going to do the math, it’s not* [referring to insufficient income], *but here they eat melon, pumpkin, pumpkin, pork* […] *then it goes.* (P8)

The impact on leisure activities was also mentioned by some family members. Such activities were restricted geographically and socially, as they were limited to the family’s residence and its surroundings. In this context, the work itself in the fields and in the care of animals ends up being constituted as leisure activities.

*She helps me, works a lot here in the fields, with animals, cuts grass* [...]. (P5)

In the next statement, the lack of adapted clothes and shoes is observed, which generated anguish and shame of the disability condition itself.

*I saw her crying, she doesn’t accept herself, she doesn’t wear short clothes to go out* [...] *or it’s pants, a long dress, a long skirt, she doesn’t want to show her legs* [...] *shoes have to match* [...] *foot is smaller, every time you go out, you have to wrap two bands and socks to be able to wear a boot.* (P12)

Regarding one of the basic rights that refers to education, DP residing in rural areas found it difficult to get to school due to access and accessibility restrictions. The long way to school was often covered on foot, due to the lack of adapted public transport.

*When she studied, access was difficult for her to catch the bus* [...] *when she had the device on her legs, we would take her to school and we would pick her up on her back.* […]. (P12)

It was also evident in the speeches that some school professionals neglected assistance to DP, which can contribute to school dropout.

*We’re sending her to class, but the teacher doesn’t do anything with the girl, she just leaves the girl there in a corner all day, so we’re not going to send her to class anymore, if that’s the case, we want the girl to go to class to learn something.* (P4)

The lack of accessibility to health services was due, among others, to the lack of transport, in addition to inadequate care and insensitivity of professionals to the life situations of DP that were reported.

*There was a time, when we went to the hospital* [...] *they didn’t pay much attention* [...] *we suffered violence from not being well assisted* [...] *to schedule an appointment at that time and the mayor says I can’t take, that’s violence too.* (P11)

Changes in the family’s life dynamics due to DP care were identified in this subcategory, in addition to financial difficulties, restriction to the rural and family environment, limitations of access and accessibility and unpreparedness of health and education professionals to care for DP. On the other hand, working with agriculture helped to alleviate the economic difficulties of families.

Chart 1 was designed to represent the synthesis of “spoken and felt violence in family, community, social and institutional contexts”.

**Chart 1:** Violence spoken and felt by disabled people, residents in rural areas, from the perspective of their families, Rio Grande do Sul, Brazil, 2022

VIOLÊNCIA FALADA E SENTIDA - contexto familiar, comunitário, social e institucional
Embarrassments and rejections Social isolation due to geographical issues in rural areas; Pejorative ways of referring to DP; Invisibility and neglect of care; Non-enforcement of basic rights; Unpreparedness of society, family members and institutional services to meet DP’s needs; Insensitivity of family, social and health network; Lack of financial resources due to the lack of a fixed salary, characteristic of rural areas; Geographical distance from rural areas; Society’s lack of adaptations to DP’s living conditions; Exclusion and restriction of access and accessibility, considering the specificities of rural areas.

*DP - disabled people.*

## DISCUSSION

As for the physical violence mentioned by participants, it affected not only the physical integrity, but also the psycho-emotional integrity of those who experienced it. A study reveals that many cases of physical violence are perpetrated by family members of DP, being considered situations of the private sphere of coexistence of families and not always discussed as a problem in rural areas, because they are treated as safe areas. The household world invisibility sometimes ends up exempting aggressors from punishment^(27)^.

Violence against DP is linked to social and gender relations, and involves the power of one over the other, almost always male over female. In rural areas, families are organized around agriculture, with a strong influence of patriarchy and social division of labor. Men work in the fields and women in the household space, taking care of family members, older adults and sick people. In this organization, there are defined and socially valued roles. When DP are born in these families, roles are tensioned, which can lead to intrafamily violence, represented by physical and verbal aggression and violation of rights.

DP face devaluation, as a result of their limitations, whether physical or intellectual, and experience prejudice and rejection by society, including their own family. In this sense, psychological violence was mentioned by participants, represented by verbal and gestural aggressions aiming to ridicule, reject and humiliate DP, isolating it from social life. Studies indicate that psychological violence corresponds to the most frequent violence, being as serious as physical violence^(28-29)^.

Experiences of disrespect, ridicule, humiliation and social discrimination are often reported by DP in their communities, resulting in shame, fear and self-stigma^(30)^. Faced with the looks, laughter and mockery of people in the community, they feel uncomfortable and hesitate to participate in sociocultural activities^(10,31)^. In this direction, it appears that DP residing in rural areas have a particularly high risk of experiencing a decrease in social participation and an increase in isolation^(15,31)^. Unfortunately, this population daily experiences negative social attitudes based on stereotypes associated with disability, significantly impacting their life experiences and opportunities and^(32)^ contributing to the invisibility of this population in the community social life^(12)^.

Stigma related to DP comes from the notion of disability anchored in the biomedical model of health, which is related to the rehabilitation of a sexed body that, for some reason, congenital or acquired, presents a deformity. Thus, socially, this body is considered different. In other words, it is a view anchored in a concept of normality of bodies. Associated with this notion are the social constructions of gender based on patriarchy and male domination present in rural areas. These visions oppress different bodies. From this perspective, it is considered that the lack of understanding of disability based on the social model contributes to situations of vulnerability of DP to various types of violence^(33)^.

The social isolation of DP due to limitations such as the rural geographic context and low social participation negatively impact the health of these populations^(34)^. In some cases, in order not to expose DP in public places, family members isolate themselves and create tense household environments, which can provoke new violence. Studies reveal that DP’s life is marked by processes of segregation and stigmatization, in which biological differences are reduced to deviation from normality. Thus, it is necessary to consider that disability is not an individual problem, but a social issue, transferring responsibility for the limitations of an individual to society, which is not able to adapt to their needs^(16,34)^.

Sexual violence was also reported by one of the family members, having been perpetrated in the presence of a family member by a health professional, while attending a female DP. Unfortunately, asymmetrical gender relations are also present in the professional-user relationship, which favors the perpetration of sexual violence. It is the duty of health professionals to ensure comprehensive health of their patients. However, in this case, the professional was the aggressor. This type of violence causes damage to individual and collective health^(35)^, and can worsen in the DP population, as many cases are not recognized as abuse, since this information comes from a marginalized group, often remaining invisibility^(36)^.

The family’s adaptations to the disability of their relative are common, because, due to the limitations of DP, they are usually dependent on their family for care^(34)^. A study reveals that strong family ties and compassion have encouraged family caregivers to change their ways of life to adapt to DP’s needs^(12)^. Families have their own ways of living, being perceived as a system that faces crises and difficulties and, in this context, family members tend to assume the role of caregivers^(37)^.

Thus, it is evident that family members also experienced restrictions and often put aside their life plans due to the care of a family member with a disability. As a result, they experienced overload situations^(38)^, being conditioned to less possibility of social mobility and economic difficulties, limited to home spaces, configuring a situation of deprivation^(16,18)^.

The impact and financial constraints are one of the common challenges faced by DP residents in rural areas and their families, since BPC is not enough to cover the expenses arising from the care that DP requires. In this way, family members need to mobilize all available resources to face critical economic conditions, seeking, for example, to produce food and raise animals for consumption. Thus, a cross-sectional study carried out in a rural area of Egypt, with 260 family caregivers of DP, revealed that 37% (n = 90) reported worsening in their economic level^(38)^. Financial difficulties prevent, among others, the purchase of medicines and the use of transport to seek health care^(13-14,31)^.

As for leisure activities carried out in rural areas, the DP and their families have few alternatives, which is conditioned to the disability characteristics and the rural areas themselves, limiting the possibilities to what the surroundings offer. With this, they dedicate themselves to leisure moments associated with household activities, farming and animal care, seeking to occupy their free time^(34)^. DP residing in rural areas live in a context in which there is a complex web of physical and socio-emotional barriers to their participation in society. For those with physical disabilities, for example, mobility is a factor of social exclusion due to the nature of their disability and the lack of assistive devices^(10,39)^.

The relevance of providing quality education to all children, regardless of any differences, is recognized by the 2030 Agenda for Sustainable Development, which, in goal 4, mentions the need to ensure inclusive, equitable and quality education^(40)^. In addition to this, the International Convention on the Rights of Persons with Disabilities and its optional protocol, in Article 24, require signatory States to guarantee equal access to quality education for children with disabilities^(41)^.

Despite this, the present study reveals that access to education by DP living in rural areas was still limited. In this sense, research, which explored the ways in which space and place influence the experience of disability lived in a rural South African context, highlighted that the education sector was another physical arena where many DP experienced exclusion, due to physical barriers and discrimination^(10)^. A study developed in Nepal, in turn, revealed that access to formal education was significantly lower for DP compared to people without disabilities, especially for those residing in rural areas^(42)^.

Research has indicated that the restricted opportunities for access to education and skills development for children with disabilities occurred due to lack of access to schools, lack of transportation and limited family income, which prevented many parents from taking their children to school^(12)^. It is identified that, despite several political guidelines worldwide mentioning the relevance of inclusive education for DP, in practice, the right to it is not fully effective, especially when particularizing rural areas.

Study points out that the rural area has more unfavorable indicators in relation to access and accessibility to health services by DP^(43)^, which is also pointed out by the present investigation. One of the impediments to this access and accessibility to health services is due to the distance of rural communities in relation to these services^(10,12-13,39)^. From this perspective, a study developed in China revealed that being a DP resident in a rural area was associated with a 13 to 40% increase in unmet needs by health services, explained, in part, by difficulties in accessing these services^(44)^. Another study indicated that the architectural design of health services was an important barrier to access and accessibility, especially for those with physical disabilities^(13)^. Furthermore, investigations revealed that there are few health services located in rural areas to meet the health care demand of DP residing in these areas, and the few that are accessible have poorly trained professionals^(31,36)^.

DP have a recognized right of access to health care, including those provided in Primary Health Care (PHC)^(41)^. In this regard, PHC can act by articulating partnerships between the education, health, justice and social assistance sectors, which are part of the Health Care Network. The health policy guideline organizes comprehensive health care based on socioeconomic, cultural, family, community and individual determinants that structure society. It is necessary to broaden the concept of violence as a problem that is not specific to health. With this, it is essential to involve and engage social actors, population, professionals and managers, with the purpose of instigating the discussion and proposing ways to guarantee health as a right^(45-46)^.

The rural context reproduces the invisibility and non-recognition of people as citizens, contributing to the lack of knowledge of needs, possibilities, access to rights and real opportunities aimed at this DP population^(47)^. Thus, it is necessary to develop strategies to expand the access of DP residing in rural areas to the rights guaranteed by national and international legislation, so that they can live in society and in less unequal conditions, compared to people without disabilities. Structural violence anchors all other forms of violence, promoting environments of exploitation and social isolation from DP, compromising their health and rights^(43)^.

### Study limitations

The limitation of this study is related to the fact that it only covered municipalities in one region of the country, which can make it difficult to generalize the results. Therefore, for future studies, the need to include other actors is indicated, aiming at a greater breadth of the investigation findings.

### Contributions to nursing, health, or public policies

By making it possible to know DP experiences in relation to this problem, even from the perspective of their families, this research brings unique contributions to the design of public policies aimed at DP residing in rural areas, in line with the Unified Health System (*Sistema Único de Saúde*) premises. Thus, it is necessary that these policies consider the double vulnerability to which this population is susceptible, related to the characteristics of the disability itself and the characteristics of rural areas and value DP’s potential and resources available in the space in which they live, although limited, as they can contribute to mitigating the reported vulnerabilities. Regarding health and nursing, the study contributes to re(thinking) professional practices aimed at this population in the Health Care Network services, considering an action based on respect, ethics and inclusion, without any forms of prejudice, violence and violation of rights.

## FINAL CONSIDERATIONS

The results show that DP residing in rural areas, according to their families, experienced situations of physical, psychological and sexual violence, perpetrated in the family, community, society and institution by both family members and colleagues, people in the community and health professionals. Moreover, they indicate that there were adaptations in family dynamics aimed at DP care, as well as financial impacts on social and leisure activities. It is also clear that DP, in this unique context, have challenges in access and accessibility to education and health services, considered basic rights of all individuals, which configures multiple situations of violence.

It can be considered that violence against DP in rural areas manifests itself in different ways, in a reality with socioeconomic and family particularities, marked by exclusion, disrespect and denial of rights and access to fundamental goods and services. Equitable access and accessibility of DP residing in rural areas, regarding education, health and leisure, can be key factors to improve this population’s quality of life, favoring their effective inclusion in society. Given the above, it is necessary to equip health professionals and other sectors of the DP care network so that they develop actions from the perspective of qualified listening, reception, inclusion and respect for human rights.
